# Medical Fees and the Public

**Published:** 1909-11-13

**Authors:** 


### EDITOR'S LETTER-BOX.

### MEDICAL FEES AND THE PUBLIC.

SIR,—Your leading article of October 23 on the above subject is unjust to medical men.

Far from fees not being lower than they should be, they are so absurdly low that it is difficult to supply patients with the costly medicines and dressings, etc., which are necessary for their treatment, and, low as these fees are, they are getting smaller still as 6d. doctors are increasing in numbers. The pay of parish doctors and club doctors is ludicrous, and medical officers of health—highly educated men who are the means of saving thousands of lives—are shamefully underpaid. And why should doctors at hospitals be expected to work like slaves for nothing, and even without thanks? Most doctors find when they get into practice that the remuneration is small and the work most arduous—their evenings, nights, and Sundays are not their own even. The fact is doctors do so much for little or nothing that they are expected to do more and are told that their fees are too high. One must take into consideration, too, that the medical profession is the most difficult and the most expensive to enter, and if things go on as they are the question will be asked, Is it worth the candle?

I am, yours faithfully,

November 9, 1909. M. R.

THE HOSPITAL November 13, 1909.

